# DNA-Directed Base Pair Opening

**DOI:** 10.3390/molecules171011947

**Published:** 2012-10-11

**Authors:** Youri Timsit

**Affiliations:** CNRS, Aix-Marseille Université, IGS UMR7256, FR-13288 Marseille, France; Email: Youri.Timsit@igs.cnrs-mrs.fr; Tel.: +33-491-825-427

**Keywords:** DNA-melting, strand separation, DNA condensation, crystal structure, base pair opening, unstacking, transcription initiation

## Abstract

Strand separation is a fundamental molecular process essential for the reading of the genetic information during DNA replication, transcription and recombination. However, DNA melting in physiological conditions in which the double helix is expected to be stable represents a challenging problem. Current models propose that negative supercoiling destabilizes the double helix and promotes the spontaneous, sequence-dependent DNA melting. The present review examines an alternative view and reveals how DNA compaction may trigger the sequence dependent opening of the base pairs. This analysis shows that in DNA crystals, tight DNA-DNA interactions destabilize the double helices at various degrees, from the alteration of the base-stacking to the opening of the base-pairs. The electrostatic repulsion generated by the DNA close approach of the negatively charged sugar phosphate backbones may therefore provide a potential source of the energy required for DNA melting. These observations suggest a new molecular mechanism for the initial steps of strand separation in which the coupling of the DNA tertiary and secondary interactions both actively triggers the base pair opening and stabilizes the intermediate states during the melting pathway.

## 1. Introduction

Although strand-separation is indispensable for fundamental genetic processes, including replication, transcription, recombination and DNA-repair, the molecular mechanisms of the initiation of DNA melting are still not well understood. Although recent crystallographic studies have revealed how replication initiators or promoter-specificity factors stabilize the open-states of DNA for replication or transcription initiation, the detailed molecular mechanisms that lead to the initiation of DNA melting remain to be elucidated [1,2]. Similarly, DNA helicases unwind DNA and translocate by a variety of mechanisms that are now well characterized, however, the first steps that trigger strand separation are still unresolved [3,4]. Indeed, while experimental and theoretical studies have shown that the nucleic acid sequence influences the stability of DNA in a complex manner, depending on both base pairing and stacking interactions [5,6,7], how to trigger DNA melting at physiological temperature in which the double helix is stable still remains an open question. Current models propose that negative supercoiling destabilizes the DNA double helix and that underwinding induces the spontaneous, sequence-dependent base flipping thus facilitating the strand separation required for various genetic functions [8,9]. 

The present review suggests an other view in which intermolecular DNA-DNA interactions may actively trigger DNA melting. Indeed, although frequently overlooked, transient or long-term DNA-DNA contacts occur in the cells and DNA crossovers represent a ubiquitous motif, at the crossroad of essential genetic functions such as DNA recombination, chromatin packaging, the control of DNA topology and the regulation of transcription and replication [10,11,12,13]. Early crystallographic studies have revealed that the assembly of double helices into tight DNA crossovers observed in crystals initiates the base-pair opening in particular sequences called “compaction responsive sequences” [13,14,15]. More recently, it has been demonstrated that the sequence-dependent assembly of DNA helices found in crystals can also occur in solution in the presence of divalent cations [16]. Moreover, right and left-handed crossovers described in crystallographic studies have been found to play an essential role in the local discrimination of the DNA topology by type II topoisomerases [17,18]. These recent findings thus reinforce the interest to investigate more deeply how close DNA-DNA interactions influence the structure and the stability of the double helix. 

Here, a survey of DNA crystal structures shows how DNA-DNA interactions induce both base pair unstacking and opening in “fragile” DNA sequences. This study reveals that the electrostatic repulsion generated by the close approach of the negatively charged sugar phosphate backbones destabilizes the double helices at various degrees, from the alteration of the base-stacking to the opening of the base-pairs, depending on their proximity and their sequences. These observations provide the structural bases for a molecular mechanism in which tight DNA-DNA interactions both actively trigger the base pair opening and stabilize the intermediate states of the melting processes by forming appropriate tertiary interactions.

## 2. Results and Discussion

### 2.1. DNA Crossovers

DNA crystals in which electrostatic repulsion between double helices is naturally minimized have unveiled helical packing modes and rules that are dictated by geometry of the DNA double helices [19,20,21]. Although each type of double helix exposes different accessible surfaces and charged groups that predispose them to interact with themselves in a different manner [22], the handedness of A- and B-double helices exerts a common constraint on their packing [15,17,18]. For example, the mutual fit of the backbone into the groove specifically generates right-handed crossovers that display particularly interesting structural and functional properties [23,24].

**Figure 1:**
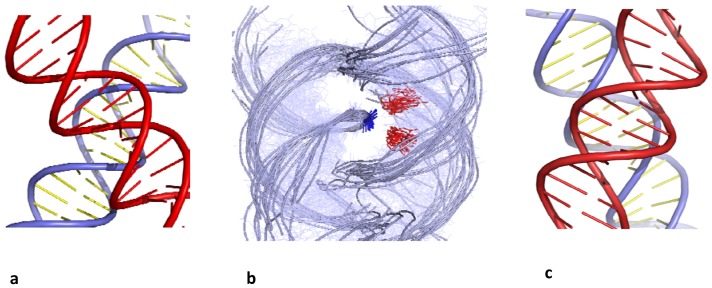
Chiral B-DNA crossovers. (**a**) Right-handed crossover assembled by the mutual fit of the backbones into the major-groove (**b**) superimposition of right-handed crossovers found in the PDB. They are all stabilized by cytosine (red) phosphate group (blue) interaction; (**c**) left-handed crossover assembled by major groove-major groove interaction.

B-DNA helices can assemble into right-handed DNA crosses by the mutual fit of their sugar-phosphate backbone into the major groove (Figure 1a,b). In contrast, due the DNA handedness, double helices can not be self-fitted in the same way within left-handed crossovers. The helices simply juxtapose their major grooves to minimize their electrostatic repulsion [25] (Figure 1c).

On the other hand, the A-form double helices preferentially self-assemble into right-handed crossovers formed by minor-groove backbone interactions. In A-form double helices, this is the shallow minor groove of the A-form that is devoted to intermolecular interactions. One of the most common elements of the ribosome structure is the interaction of RNA double helices via minor grooves [26]. Inter-helical packing involving minor-groove backbone interactions have been observed in the crystal packing of many RNA oligonucleotides [27,28] and constitutes a common packing motif that has been also observed within the structure of the ribosomal subunits [29]. DNA sequence plays a different role for the packing of A- and B-DNA helices. Indeed, a comparison of DNA crystal packing modes revealed that the interactions between A-DNA helices are much less dependent from the DNA sequence than the B-DNA ones [21].

**Figure 2:**
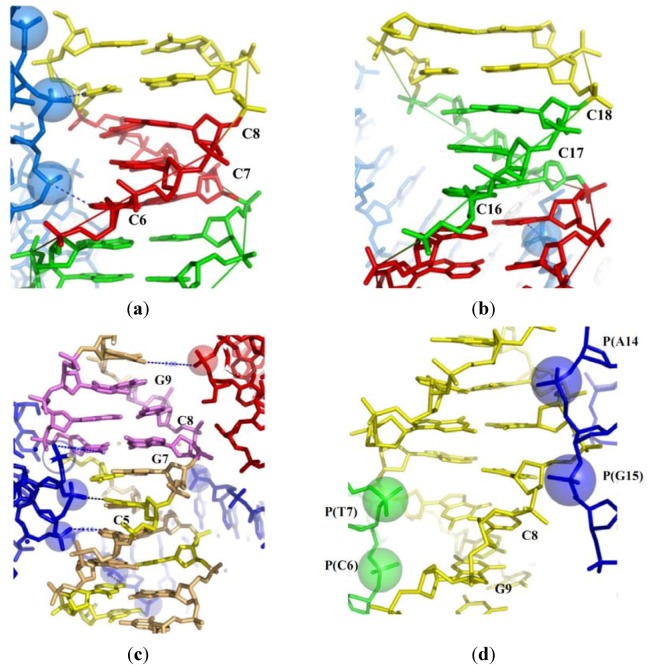
Packing-induced base stacking alterations. (**a**) The half of the palindromic decamer d(CCIIICCCGG) (I denotes an inosine) exposed to three phosphate groups of a symmetry related molecule that form a left-handed cross; (**b**) The free half of the decamer; (**c**) the structure of the decamer d(CCGCCGGCGG) surrounded by the three symmetry related molecules; (**d**) The structure of the decamer d(CCGAGCTCGG) showing the backbone of two symmetry related molecules deeply inserted in the minor groove.

Right-handed crossovers of B-DNA double helices are unique in that they are assembled by a sequence-dependent interaction. While the B-DNA double helix dictates the geometry of inter-helical assembly, cytosines play a key role for controlling the interaction through specific interaction of their N4 amino groups with phosphate groups [23,24,30]. Remarkably, most of the right-handed crosses examined to date are assembled by the major groove-backbone interaction, involve cytosine-phosphate group interaction at the anchoring point (Figure 2b) and are frequently stabilized by divalent cations. A recent survey of crossover structures has shown indeed that, without exception, cytosine-phosphate interactions are strictly required for stabilizing right-handed DNA crossovers. Probably due to the vicinity of the N7 group that displays a negative potential, the N6 amino group of adenine does not substitute the N4 amino group of cytosine for this type of interaction [31]. In addition, crystallographic studies of methylated DNA duplexes showed that C5-methyl cytosines also promote the formation of DNA crossovers at the modified C5-mpG sequences [32]. The two methyl groups form a hydrophobic clamp which traps the incoming phosphate through C–H…O interactions that further stabilize the helical assembly.

The free energy of interactions of DNA duplexes in right and left-handed crossovers as a function of divalent cation concentration in solution has been investigated using molecular dynamics simulations [16]. This study showed that right-handed DNA crossovers (Figure 1a) are thermodynamically stable in solution in the presence of divalent cations. Consistent with recent theoretical and experimental observations of close DNA-DNA interactions in the presence of divalent cations [33,34,35], a short-range attraction of about −4 kcal·mol^−1^ between the self-fitted duplexes was observed in the presence of divalent cations [16]. Right-handed crossovers, however, dissociate in the presence of monovalent ions only. Consistent with the crystallographic studies, molecular dynamics simulation showed that two helices remain assembled by specific cytosine-phosphate interactions and bridging Mg^2+^ ions at the duplex interface. The repulsion of the negatively charged backbone is circumvented both by the specific relative orientation of helices and by the presence of Mg^2+^. Therefore, similar structural features of the right-handed crossovers are present in solution and in the crystal environment. Four-way junctions and right-handed crosses share an analogous geometry that is stabilized by similar tertiary interactions involving cytosines and Mg^2+^ [23,36]. The folding of particular RNA motifs found in many functional RNA molecules also requires specific divalent cations [37,38]. A common feature in most of these structures is the anchoring of a phosphate group to a guanine base through a divalent cation bridge.

In contrast, left-handed crossovers are unstable at similar ionic conditions and resulted in a swift dissociation of the helices. With a less clear pattern of specific intermolecular interactions, left-handed helix juxtapositions by major groove-major groove interaction (Figure 1c) are stable only in the crystallographic environment but appeared to be unstable in solution.

Overall, these works have put in light the particular role of cytosines for controlling spatial organisation and the stability of tertiary DNA assemblies. The formations of inter or intramolecular H-bond between the N4 amino group of cytosine and a phosphate group plays therefore a key role for controlling DNA-DNA interactions in a sequence dependent manner. 

### 2.2. Tight Contacts in DNA Higher-Order Structures and Supercoiling

DNA-DNA interactions that occur during DNA condensation [39] have been also found to play a critical role in DNA topology and in the building of higher-order DNA structures. For example, within the interwound plectonemic supercoiled DNA that play a key role in gene regulation in both prokaryotes and eukaryotes [9,40], closely packed regions with intersegmental contacts occur, in the presence of divalent cations, under physiological conditions [41,42,43]. The importance of such close contacts has been also noted for the knotting of supercoiled DNA [44]. Type II topoisomerases play a major role in disentangling sister chromatids during replication and in maintaining the fine balance of superhelical density [40]. Crossover geometry and stability has been demonstrated to be essential for the local sensing of DNA topology by type II topoisomerases [17,18].

Chromatin folding also involves close interactions between the linkers or nucleosomal DNA [45]. DNA crossovers self-fitted by groove-backbone interaction have been observed in the crystal packing of many nucleosome structures [46] and close DNA-DNA interactions are seen in the recent all-atom model of the chromatin fiber [47]. Moreover, the stability of right-handed crossovers in physiological conditions supports earlier hypotheses that groove-backbone fitting organizes the geometry of nucleosomal or linker assembly within the chromatin fibre [23,24]. Therefore, the cell disposes of a collection of direct DNA-DNA interactions with varying degree of stability that can be exploited for tuning chromatin compaction and controlling DNA topology.

### 2.3. DNA-Directed Base-Pair Opening

In addition to direct the organisation of higher-order DNA structures, DNA-DNA contacts may also influence the double-helix structure and stability. Soon after the first X-ray studies on single-DNA crystals, the question of the effect of the crystal packing on DNA structure has been investigated, showing that a complex and reciprocal link relates DNA structure and packing forces [48]. Since intermolecular interactions observed in crystals also occur in solution, the packing contacts provide very useful information about the coupling between tertiary interactions and secondary structure of DNA that can participate in molecular processes *in vivo*.

#### 2.3.1. Close Helical Approach and Base Stacking Alterations

Firstly, a survey of B-DNA structures within a set of closely packed DNA crossovers [17,31] shows that the marked irregularities of base-stacking are induced by the close approach of the negatively charged backbones of neigbouring molecules. This effect is clearly visible in comparing the structures of two independent halves of a palindromic duplexes (in which the palindromic axis does not fit with a 2-fold crystallographic axis). For example, Table 1 shows that the alterations of the base stacking in one of the two halves of the decamer duplex d(CCIIICCCGG) (bdjb77) correlate with the proximity of the phosphate groups of symmetry related molecule. The insertion of the phosphate groups P(10) and P(9) of a neighbouring molecule at 7.5 and 8.1 Å of the helical axis significantly increases the value of buckkle and propeller twist of the base pairs from C6-I15 to C8-I13 [25] (Table 1; Figure 2a). In contrast, in the equivalent duplex half (from I3-C18 to I5-C16) that is not exposed to the phosphate groups of equivalent molecules, the geometry of the base-pairs is much more regular (Table 1; Figure 2b).

**Table 1:** Base stacking alterations induced by DNA-DNA interactions. The distances between the phosphorus atoms of the symmetry related molecules and the helical axis, in front of the corresponding base-pairs are written in red.

Bdjb77				Bd0015				Bd0084			
	Prop.	Buckle	Dist.		Prop.	Buckle	Dist.		Prop	Buckle	Dist.
	(°)	(°)	P-axis		(°)	(°)	P-axis		(°)	(°)	P-axis
			(Å)				(Å)				(Å)
**C1-G20**	−5.2	10.2		**C1-G20**	−8.7	6.8	P(15)	**C1-G20**	−11.6	−0.4	
							**8.2**				
**C2-G19**	0.6	1.4		**C2-G19**	−9.9	−6.6	P(14)	**C2-G19**	15.6	1.4	
							**6.6**				
**I3-C18**	−10.1	−0.5		**G3-C18**	−14.5	−12.2		**G3-C18**	−7.0	1.4	
**I4-C17**	−6.8	−2.6		**C4-G17**	−30.6	1.8	P(18)	**A4-T17**	5.7	−3.3	
							**8.1**				
**I5-C16**	−12.4	1.9		**C5-G16**	− 9.2	6.5	P(17)	**G5-C16**	−24.5	−5.4	P(A14)
							**6.9**				9.8
**C6-I15**	−15.2	−0.7	P(10)	**G6-C15**	−11.4	−1.8		**C6-G15**	−11.0	1.2	
			**7.5**								
**C7-I14**	−15.3	11.6		**G7-C14**	−26.1	0.3		**T7-A14**	−5.8	2.0	
**C8-I13**	−13.6	16.8		**C8-G13**	−14.0	−3.5		**C8-G13**	−12	2.0	P(T7)
											**6.8**
**G9-C12**	−6.1	4.8	P(9)	**G9-C12**	−28.7	3.3		**G9-C12**	−23.3	−23.4	P(C6)
			**8.1**								**8.0**
**G10-C11**	−17.3	1.6		**G10-C11**	−16.8	−4.7		**G10-C11**	−14.9	6.3	

Although containing exclusively G-C base pairs, the decamer duplex d(CCGCCGGCGG) (bd0015) displays a highly distorted structure. The anomalies in base stacking characterized by extreme values of buckle and propeller-twist (Table 1) can also be explained by the deep insertions of phosphate groups from three symmetry related molecules into the major groove [24] (Figure 2c). Two sets of consecutive cytosines (C1, C2 and C4, C5) bind two phosphate groups (P15, P14 and P18, P17), respectively, through their N4 amino groups. This crystal structure that represents, to my knowledge, one of the most condensed packing of B-DNA double helices observed to date, clearly illustrates how the electrostatic repulsion may alter the base-stacking and thus affects the stability of the double helix even in a DNA sequence exclusively composed of GC base pairs. In contrast, the same decamer d(CCGCCGGCGG) adopts a quite regular structure when crystallized in the A-form in a totally different electrostatic environement [32].

A third example of pronounced alteration of base-stacking is observed in the structure of the decamer d(CCGAGCTCGG) (bd0084) [49]. The packing provides a rare example of left-handed crossover juxtaposed by minor groove-minor groove interaction (Table 1). In this case, the phosphate groups of the symmetry related molecules are deeply inserted into the minor groove. They are localized in front of the N2 amino groups of guanines, that generate a positive potential along the minor groove surface, similar to the N4 amino groups of cytosine in the major groove. As seen in Table 1 and Figure 2d, the presence of the four negatively charged phosphate groups P14, P15, P6 and P7 of two neigbouring duplexes at close proximity of the base-pairs C8-G13 and G9-C12 contributes to destabilize their stacking interactions. These three examples show how the close approach of B-DNA double-helices mutually alters their base-stacking geometry thus contributing to destabilize the DNA structure.

#### 2.3.2. Close Helical Approach and Base Pair Opening

Intermolecular contacts may also induce both base-pair unstacking and unpairing. Because of their intrinsic instability, these structures are however rarerely observed and represent a few exceptional cases of “trapped intermediates” reported in the PDB. Two types of such structures can be distinguished. In the first one, the phosphate group inserted into the major groove *directly* breaks a GC base pair by pulling-out the cytosine. In the second case, the inserted phosphate group binds the N4 amino group of a cytosine without disrupting the GC base pair, but it *indirectly* unstacks and opens the neighbouring base pairs (most frequently AT base pairs).

##### 2.3.2.1. Direct Base-Pair Opening

**Figure 3:**
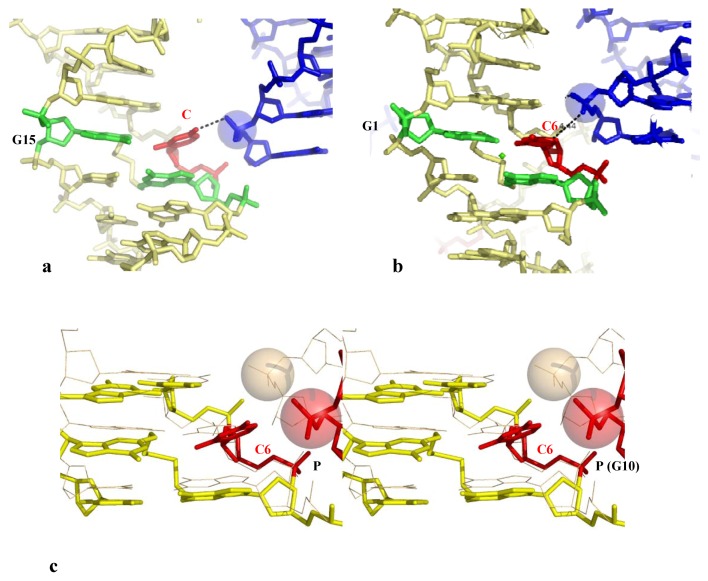
Direct Base pair opening induced by DNA-DNA interactions. (**a**) The open form of the decamer d(CCAGGCCTGG) (the G denotes the crosslinked G); (**b**) The closed form of the same decamer. The flipped out cytosine in the open form and the corresponding one in the closed one, are represented in red;(**c**) Stereo view of the superimposition of the open (yellow, thick line) and closed (wheat, thin line) forms with their symmetry related molecule. The opening phosphate group is represented in red.

A clear demonstration of the direct opening of a GC base pair induced by a phosphate group of a symmetry related molecule is provided by the high resolution structure of the crosslinked decamer duplex d(CCAGGCTGG)_2_, (G denotes the ethyldisulfide crosslinked guanines) [50]. This decamer crystallizes with two distinct molecules in the asymmetric unit, one having a canonical duplex whereas the other displays a distorted DNA structure and an open GC base pair. Interestingly, the open form clearly correlates with the presence of an inserted phosphate into the major groove at an appropriate position for pulling-out and stabilizing the flipped-out cytosine. The base pair C6-G5 is partially ruptured, with two broken N3-N1 and N4-O6 Watson-Crick interactions, for forming a tertiary H-bond N4 (C6)-O1P (G10#) with the symmetry related molecule (Table 2, Figure 3a).

**Table 2:** Direct and indirect base pair opening induced by DNA-DNA interactions. The star denotes the cytosine that makes an hydrogen bond with the inserted phosphate group.The # denotes a residue from a symmetry related molecule. The base-pairs represented in red are partially disrupted.

	Space group	Resol.	Sequence	Structural alteration
**Base unstacking**				
Bdjb77 (286D)	P3_1_	2.5	5'-CC I I I CCCGG	High propeller twist and buckle values (from C6-I15 to C8 I13) in front of inserted phosphate groups (Table 1)
			3'-GGCCC I I I CC	
Bd0015 (1QC1)	H 3	2.5	5'-CCGCCGGCGG	Alterations of base stacking along the whole structure (Table 1)
			3'-GGCGGCCGCC	
Bd0084 (1ZFG)	P3_1_	1.75	5'-CCGAGCTCGG	Alterations of base stacking in front of the inserted phosphate groups (Table 1)
			3'-GGCTCGAGCC	
**Direct base-pair opening**				
Bd0022 (1QP5)	H 3	2.6	* *	C3-G17(#): N4-O1P: 2.4 Å
				C21-G17(#): N4-O1P: 3.3 Å
			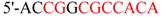	C18-C20(#): N4-O1P: 2.9 Å
			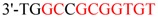	
			* *	Shift of the base pairing in the major groove (Figure 5) from 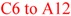
Bdl035 (330D)	H 3	2.7	5'-ACCGCCGGCGCC	Normal base-pairing
			3'-TGGCGGCCGCGG	
Bd0076 (1ZEW)	C2	2.25	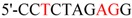	C12-G7(#): N4-O1P: 2.7 Å
				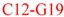 : N4-O6: 4.0 Å
				N3-N1: 3.3 Å
				O2-N2: 2.7 Å
			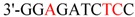	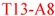 : O4-N6: 3.6 Å
			*	N3-N1: 3.6 Å
				A18-T15(#) N6-O1P: 6.9Å
				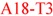 : N6-O4 : 4.0 Å
				N1-N3: 3.7 Å
Bd0028 (1ZFH)	C2	2.5	5'-CCGCTAGCGG	Normal base-pairing
			3'-GGCGATCGCC	
Bd0021 (1CW9)	P2_1_2_1_2_1_	1.55	*	C6-G10(#): N4-O1P: 3 Å
			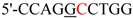	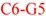 : N4-O6: 7.0 Å
			3'-GGTCCGGACC	(2 mol/au: open and closed form)
**Indirect base-pair opening**				
Bdf062 (206D)	P6_1_22	2.5	*	C1-C8(#): N4-O1P: 3.3 Å
				C10-A9(#): C6-O1P: 2.9 Å
			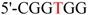	C5-O2P: 3.3 Å
				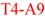 : O4-N6: 4.7 Å
			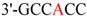	O2-N6: 2.9 Å
				Unstacking C1/G2/G3
Ud0029 (1P4Z)	P6_1_22	2	*	C7-A6(#): N4-O1P: 3.0 Å
			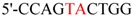	N4-O6: 3.3 Å
			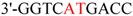	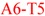 N6-O4: 3.8 Å
			*	N1-N3: 3.5 Å
Bd0068 (1S1K)	P6_1_22	1.9	5'-CCMGTACTGG	Normal base-pairing
			3'-GGTCATGMCC	
			M=1AP	

In contrast, in the closed-form, the symmetry related molecule occupy a different position that does not make possible the formation of an equivalent H-bond between the phosphate group and the C6 cytosine (Figure 3b,c). The phosphate group is located in front of the C7 cytosine that is slightly rotated for forming a C–H...O interaction C5 (C7)...O1P (G10) without breaking its W-C interaction with its complementary guanine (Figure 3c). In addition a heptacoordinated calcium ion sits in front of the two G15 and G5 guanines and therefore contributes to attenuate the electrostatic repulsion generated by the mutual approach of the negatively charged sugar-phosphate backbones (Figure 3b). Thus, the comparison of the electrostatic environements around the two distinct molecules of the asymmetric unit leads to a description of how the position of the inserted phosphate in the major groove is critical for disrupting the GC base-pair.

**Figure 4:**
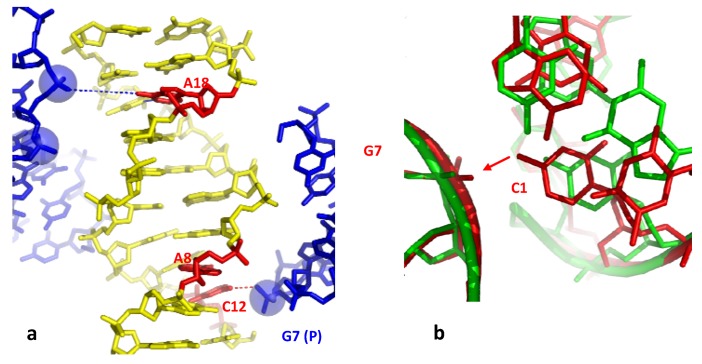
(**a**) The decamer d(CCTCTAGAGG) surround by two symmetry related molecules. The open base-pairs are represented in red; (**b**) Superimposition of the decamer d(CCTCTAGAGG) (red) that displays open base-pairs and the decamer d(CCGCTAGCGG) that displays a normal structure (green).

A second example of the direct opening of a GC base pair by a phosphate group is observed in the crystal structure of the decamer d(CCTCTAGAGG)_2_ (Table 2) [51]. Within the right-handed crossovers formed by two symmetry related DNA molecules, the phosphate group of one duplex has partially broken the base pair C12-G19 and its immediate neighbour T13-A8 (Table 2). Similar to the previous example, the cytosine C12 preferentially interacts with the inserted phosphate group of G17 rather than to remain fully paired with its complementary guanine G7. The partial opening of the base pair is also accompanied by marked irregularities of the base stacking geometries that propagates to the close neighbour T13-A8 base pair which is both unstacked and opened (Table 2 and Figure 4a). Four nucleotides away, the distorted structure and the partial disruption of the base pair A18-T3 also correlates with the presence of a phosphate group in front of the A18 adenine. Although too far for forming a H-bond with its N6 amino group of A18 (6.9 Å), it interacts through a C–H...O interaction with the C5 atom of the C2 cytosine (Table 2). A comparision of the structure of d(CCTCTAGAGG)_2_ (bd0076) with four isomorphous decamer duplexes crystallized in the same C2 space group reveals that packing induced structural distortion is sequence dependent. Indeed, in the three isomorphous decamer duplexes bd0028, bdj060 and bdj081, although the equivalent C12 cytosine forms a hydrogen bond with the inserted phosphate group it remains paired with its complementary guanine. Remarkably, the substitution of a single base pair, from T-A to G-C in the decamer d(CCGCTAGCGG)_2_ (bd0028) restores a stable structure with a normal pairing and stacking that seems “unresponsive” to intermolecular contacts. It could be therefore deduced that a thymine located immediately in 3' of an anchoring cytosine fragilises the double helix that becomes more easily destabilized by the DNA-DNA contacts. The superimposition of the two isomorphous structures (bd0076 in red and bd0028 in green) shows that whereas in bd0076, the phosphate group is slightly more deeply inserted in the major groove than in bd0028, the structural alterations of bd0076 come mainly from the reorganisation of the bases around the anchoring point (Figure 4b). 

In particular, the translation of the cytosine C12 towards the inserted phosphate group fully unstacks it from its 3'-neighbour T13. Reciprocaly, it can be rationalized that the increased deformability of the C12-G19 base pair from which C12 is pulled-out by the inserted phosphate group, depends on the high flexibility of the CT step (C12-T13). Cytosines comprised within a CT step are therefore more prone to be unpaired by intermolecular contacts.

**Figure 5:**
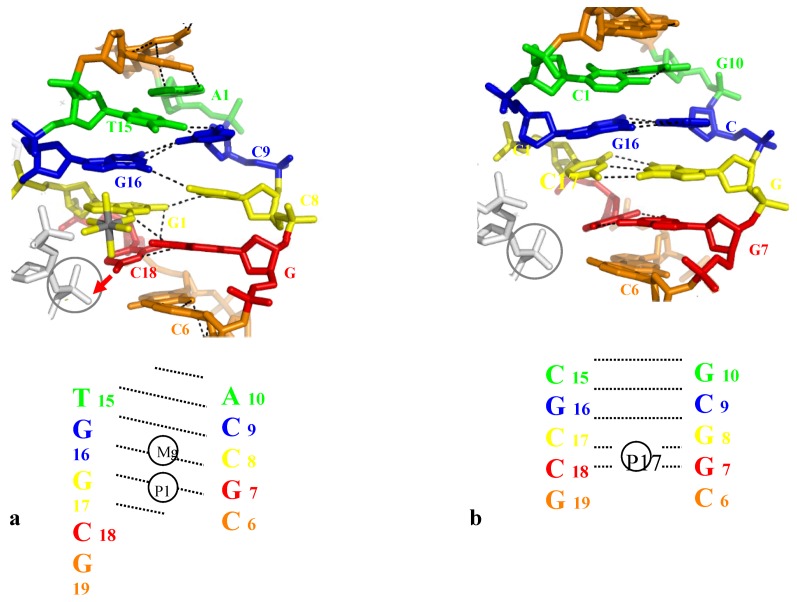
(**a**) Shift of the base pairing in the dodecamer d(ACCGGCGCCACA). The backbone of the fitted molecule is represented in grey. The bases that should be normaly paired are represented with the same color; (**b**) The corresponding region of the dodecamer d(ACCGCCGGCGCC) that displays a normal base paring.

An third example is provided by the temperature sensitive crystals of the B-DNA dodecamer d(ACCGGCGCCACA). d(TGTGGCGCCGGT) (bd0022) that has been solved twenty years ago [14,15]. This structure showed that, in pulling-out the interacting cytosine, the phosphate group inserted into the major groove has not only broken the corresponding G-C base pair but has also propagated a dramatic rearrangement of the base pairing, on both sides of the packing anchoring cytosine (Table 2 and Figure 5a). These marked structural alterations that are correlated with the great thermal sensitivity of the crystals suggested that the low temperature crystals had captured a “premelted” state of the double helix [14,15]. Although submitted to identical intermolecular contacts, the structure of the duplex d(ACCGCCGGCGCC). d(GGCGCCGGCGGT) (bdl035) has been found to be nearly unaffected by the packing contacts and the crystals were stable up to 37 °C (Table 2, Figure 5b).

It has been concluded that the unique properties of bd0022 depends on its sequence that first, contains a set of the most unstable dinucleotide step in term of stacking (CpA or TpG) and second, that has the potential to accomodate the concerted shift of the base pairing along the major groove (Figure 5a). The particular electrostatic partition of the two DNA strands, consisting of one strand with a set of major groove donor bases (C/A)*_n_* (amino groups N6 or N4 of A or C) and another one with a set of major groove acceptor bases (T/G)*_n_* (carbonyl groups O6 or O4 of G or T), is suitable for rearrangement, in a domino-like motion, of the base pairing. In summary, depending on the sequence and the mode of phosphate insertion, the direct opening of a GC base pair may either remain local or propagate to the neighbouring base-pairs. 

##### 2.3.2.2. Indirect Base-Pair Opening

**Figure 6:**
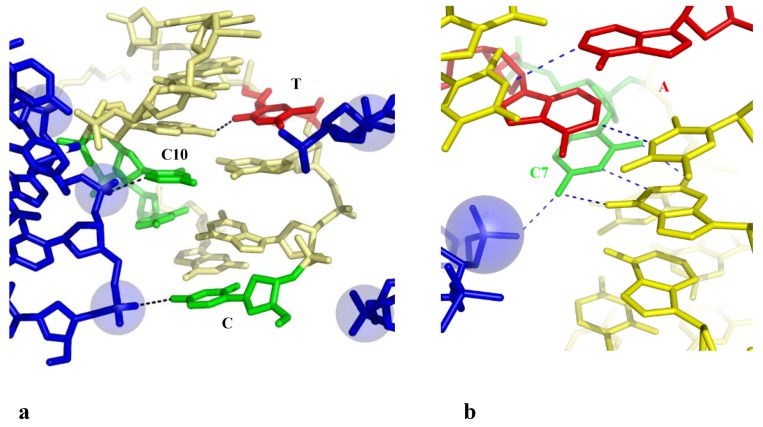
Indirect base pair opening induced by DNA-DNA interaction. (**a**) The hexamer d(CGGTGG) surrounded by two symmetry related molecules. The open base pairs are represented in red; (**b**) Detail of the groove-backbone interaction in the decamer d(CCAGTACTGG).

Two examples reveal that a phosphate group that binds a cytosine without breaking the G-C base pair, may indirectly disrupt its immediate neighbours in specific sequences. The structure of the hexamer d(CGGTGG). d(CCACCG) displays a highly irregular helical geometry with several unstacked bases and an open AT base pair (T4-A10) (Table 2 and Figure 6a) [52]. The analysis of the packing contacts shows that these structural alterations are, once again, linked to the presence of many negatively charged phosphate groups of symmetry related molecules into the major groove. The C1 and C10 cytosines that interact through H– or C–H...O bonding with two of them are fully unstacked with the surrounding bases and the resulting structure is so distorted that the T4-A9 base is almost fully open, with only one rearranged interaction between the O2 (T4)-N6 (A9). 

Another example of indirect base pair opening is observed in the highly distorted structure of the decamer d(CCAGTACTGG)_2_ in which the two central AT base pairs are open (Table 2) [53]. Figure 6b shows that the disrupted AT base pairs are also the immediate neighbours of a cytosine (C7) that is hydrogen bonded to the phosphate group of a symmetry related molecule. In contrast, the isomorphous crystal structure of the decamer d(CC(1AP)GTACTGG) (where 1AP denotes aminopurine) that only differs by the presence of an additional NH_2_ group in the minor groove side of A3, displays a regular structure [53]. Thus, a single additional W-C interaction that further stabilise the base pair on one side of the anchoring cytosine appears to be sufficient for preventing the partial opening of the base pair on the other side. Subtle changes in the sequence may therefore modulate the double-helix sensitivity to the intermolecular contacts. In addition, as observed in the closed-form of the decamer d(CCAGGCCTGG)_2_ (see above), a Ca^2+^ ion that bridges the N7(A6), to the inserted phosphate, likely contributes to stabilise the structure in shielding the electrostatic repulsion. This ion is not observed in front of the open A6-T5 base pair of the decamer d(CCAGTACTGG)_2_.

These examples demonstrate that the duplex sequence, the geometry of the intermolecular interaction and the presence of divalent cations modulate the structural response to DNA-DNA interactions. The alterations induced by the close helical approach vary from the disruption of a single base-pair to the induction of a premelted structure depending on the sequence and electrostatic context. The comparison of the isomorphous structures reveals that the sequences that contain highly flexible CA, TC and TA steps [6,54] at the vicinity of the anchoring cytosine are deformed by the penetration of the phosphate group into the major groove while the other duplex structures remain unaltered. Sequences that have the potential to rearrange base-pairing such as (C/A)*_n_* or (T/G)*_n_* are able to propagate the local deformation and to stabilise a “premelted” state of the double helix.

### 2.4. A Model for the Initiation of DNA Melting Induced by Compaction

The problem of DNA melting at physiological temperature is to find a source of energy for triggering base-pair opening. A current view is that torsional stress is a potential source of the energy that extrudes bases from the helix [8,9]. The present study reveals that the electrostatic repulsion generated by the DNA compaction may provide another potential source of the energy required for base flipping and DNA melting. Intermolecular interactions in crystals have brought useful insights about biologically relevant tertiary interactions and may represent possible interaction modes for DNA higher-order structures *in vivo*. Indeed, it has been recently shown that the stable DNA crossovers observed in crystals also occur in solution, in the presence of magnesium ions at concentrations close to physiological conditions [16]. Also, many tight inter-helical interactions observed in large RNA structures are reminiscent to that found in oligonucleotide crystals [26,29].

This review therefore suggests a molecular mechanism in which the close DNA-DNA intermolecular interactions that occur in genome packaging, DNA looping or supercoiling may initiate, in a sequence-dependent manner, the strand separation required for the reading of the genetic information. 

**Figure 7:**
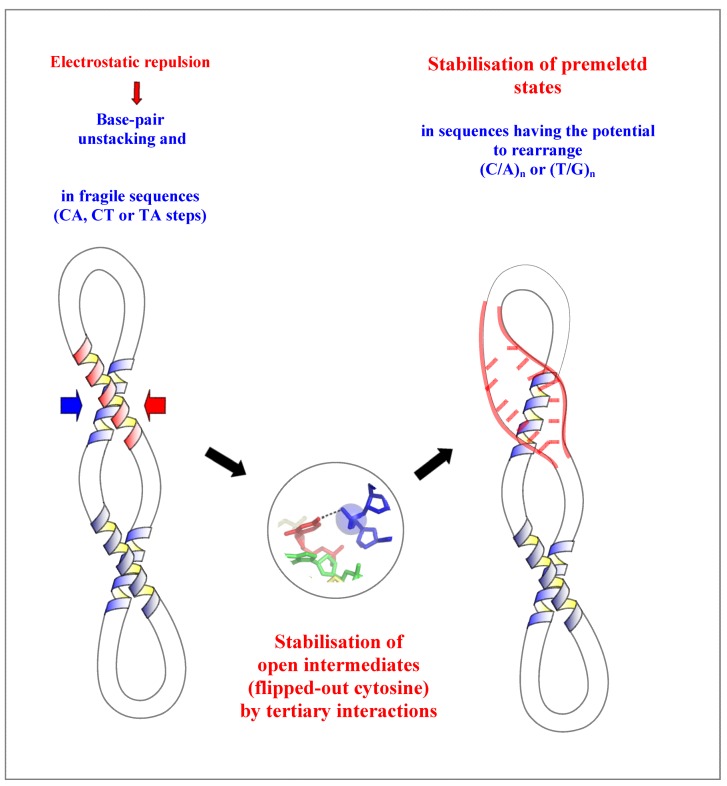
Model of a molecular mechanism of DNA-directed base pair opening.The close approach of two DNA segments in supercoiled DNA or higher-order structure can induce the initiation of strand separation in specific sequences such as (C/A)_n_ or (T/G)_n_ represented in red.

The model proposes first, that the electrostatic repulsion due to the close approach of the negatively charged backbones alters the base stacking and destabilizes the helix. This contributes to increase the base breathing in DNA sequences containing flexible CT, TG or TA steps. Second, a phosphate group of the neighbouring DNA segment both pull-out a cytosine and stabilizes it into a flipped-out conformation. This further destabilizes the secondary structure of the duplex that will rearrange readily in sequences that have the potential to form alternative pairing such as the (C/A)*_n_* or (T/G)*_n_* tracts. The rearranged structure of higher energy may be therefore considered as an intermediate “premelted” state of the DNA (Figure 7). The importance of these of pre-melted intermediates in the DNA melting pathway has been point out in a recent molecular dynamics study. During the melting pathway, the DNA strands slide and search the local base pairing by alternately forming bifurcated and W-C H-bonds [55], as observed in the dodecamer d(ACCGGCGCCACA) (bd0022) [14].

Thus, the coupling between tertiary and secondary DNA structure in DNA crossovers acts as a “catalyst” that stabilizes a set of intermediate steps (flipped-out base and rearranged base pairs) of higher energy (transition state) in the pathway of DNA melting. Such coupling between tertiary and secondary interactions play also a crucial role during the folding of RNA [38].

Although nucleosomal DNA is expected to be stabilized by its interaction with the histones, DNA melting may be potentially triggered by the crossing of the linkers at specific sequences. Proteins that promote the formation of crossovers or DNA looping such as topoisomerases, recombinases and H1 histones may therefore indirectly assist DNA-directed base-pair opening in *compaction responsive* sequences that are frequently found in promoter, replication origin and hotspots of recombination [13].

## 3. Conclusions

This short review shows firstly, that the double helix geometry and handedness play a critical role in the building rules of DNA higher-order structures. Secondly, the close DNA-DNA interactions can also trigger strand separation in specific sequences. This study shows that in its condensed state, DNA possesses all the attributes to behave as a catalyst that stabilizes and lowers the energy of the transition states during the pathway of DNA melting. Supercoiled or compacted states of DNA have therefore the potential to specifically initiate DNA melting and propagate strand-separation through slithering or other kinds of motions between DNA segments occuring *in vivo*. Compact DNA structures may be therefore compared to a helicase or other enzymes that catalyze the flipping out of the bases. These observations provide alternative models for understanding mechanism DNA unwinding required in numerous genetic functions such as replication, transcription and recombination.
